# Editorial: The role of neutrophil extracellular traps formation in tumor microenvironment – from basic research to clinical applications

**DOI:** 10.3389/fimmu.2026.1812937

**Published:** 2026-03-10

**Authors:** Rachel Shukrun Zicherman, Alexey Stepanov, Xue-Yan He, Ronit Elhasid

**Affiliations:** 1 Pediatric Hemato-Oncology Research Laboratory, affiliated with the Gray School of Medicine, Tel Aviv University, Tel Aviv, Israel; 2 The Rockefeller University, New York, NY, United States; 3 Department of Integrative Structural and Computational Biology, The Scripps Research Institute, La Jolla, CA, United States; 4 Department of Cell Biology and Physiology, School of Medicine, Washington University in St. Louis, St. Louis, MO, United States

**Keywords:** biomarkers, cancer immunology, neutrophils extracellular traps (NETs), therapeutic targets, tumor microenvironment

Neutrophil extracellular traps (NETs) are web-like structures composed of chromatin decorated with histones, proteases, and antimicrobial proteins that are released by activated neutrophils. They were first described as a specialized antimicrobial defense mechanism, but it is now clear that dysregulated NET formation contributes to autoimmunity, thrombosis, organ damage, and cancer. In tumors, NETs accumulate both within the microenvironment and in the circulation, where they can entrap circulating tumor cells, remodel the extracellular matrix, modulate immune cell function, and promote metastasis and treatment resistance.

Many clinical studies across different malignancies have demonstrated that NETs and NET-associated markers carry prognostic information in sizable patient cohorts. In this Research Topic, a large systematic review and meta-analysis (Lee et al.) of tissue and circulating NET biomarkers in cancer synthesizes data from 5,202 patients and shows that higher NET levels, measured in both tumor tissue and blood, are consistently associated with poorer overall and disease-free survival, whereas cell-free DNA alone is not prognostic. Several contributions explore organ and disease specific NET landscapes. A review on NETs in diseases of the female reproductive organs (Morawiec et al.) surveys NET involvement in breast, ovarian, cervical, and endometrial cancers, as well as in premature ovarian insufficiency, cervicitis, endometriosis, pregnancy, and pregnancy-related complications. Across these conditions, elevated NETs or NET-derived markers are frequently associated with advanced stage, metastasis, thrombosis, and adverse pregnancy outcomes, and NETs are interwoven with EMT, immune escape, and tissue damage. From the viewpoint of individual cancers, a minireview on tumor-associated neutrophils (TANs) in renal cell carcinoma (RCC) (Kovaleva et al.) highlights how tumor-associated neutrophils and NET-forming subsets participate in angiogenesis, T-cell exclusion, and resistance to immune checkpoint inhibitor/tyrosine-kinase inhibitor combinations and describes RCC-specific TAN/NET signatures that may refine risk stratification. Another contribution, an integrated multiomics and machine learning analysis of NET-related molecular subtypes in acute myeloid leukemia (Zhong et al.) across nine cohorts, uses multi-cohort transcriptomic data to define NET-related molecular subtypes and a 22-gene NET-based risk score that stratifies patients by outcome, immune contexture, and likely response to chemotherapy and PD-1–based immunotherapy. Collectively, these studies highlight the clinical significance of NETs across malignancies and illustrate how NET-related biomarkers and gene signatures can be embedded in risk models and therapy-selection frameworks.

A major advancement in the field of NETs has been the detailed characterization of mechanisms through which NETs affect cancer and stromal cells. A minireview on NETs as drivers of epithelial-mesenchymal transition in cancer cells (Maddalena et al.) focuses on the ability of NETs to drive epithelial–mesenchymal transition (EMT), summarizing data from endothelial, breast, lung, gastric, colorectal, and pancreatic models. NET-associated DNA and proteases trigger loss of epithelial markers, gain of mesenchymal markers and EMT transcription factors, and increased motility and invasion via multiple pathways, including β-catenin, TGFβ/Smad, NF-κB/NLRP3, Notch1, and the CCDC25–integrin–ILK axis. While most studies indicate that NETs promote cancer progression and therapy resistance, some studies suggest that NETs may have an anti-tumor effect. Interestingly, an original study of human neutrophils from lung-cancer patients (Alsharif et al.), showing that lung cancer neutrophils generate NETs with preserved anti-tumor cytotoxicity but impaired anti-migratory activity, demonstrates a qualitative rewiring of NET functions in cancer: neutrophils from patients lose the anti-migratory effect of their NETs on tumor cells, so that patient-derived NETs, while still tumoricidal, can now promote tumor cell motility. This duality highlights the importance of defining when and where NETs are pathogenic versus protective within distinct tumor microenvironments.

The metabolic and microenvironmental control of NETs is another key focus of this Research Topic. An *in vitro* study of HIF-1α-dependent glycolysis in hypoxia-induced NETosis (Ye et al.) shows that hypoxia robustly induces NET formation in primary neutrophils and neutrophil-like cell lines via HIF-1α–dependent glycolysis. Hypoxic stimulation stabilizes HIF-1α, upregulates glycolytic enzymes and PADI4, increases citrullinated histone H3, and enhances NET release and neutrophil elastase secretion, whereas pharmacologic inhibition of HIF-1α or GLUT1 reverses these effects and markedly reduces NETosis. These findings tie NET formation to the metabolic state of hypoxic tumor microenvironments and provide a mechanistic rationale for targeting metabolic drivers of NETosis in cancer. An experimental study in equine innate immunity (Salinas-Varas et al.), showing that equine adipose-derived stem cells modulate *in vitro* NET release by polymorphonuclear neutrophils, while outside oncology, shows that equine adipose-derived stem cells can dampen NET formation by neutrophils without altering reactive oxygen species production. This work illustrates how stromal-like cell types can directly tune NET formation, a theme that resonates with the complex cellular crosstalk within tumor microenvironments.

Finally, this group of studies also addresses the potential of NETs as a therapeutic target. A second review, focusing on NETs as predictors and targets of supportive therapy for cancer treatment (Morawiec et al.), extends this perspective across tumor types and catalogs NET roles in carcinogenesis, metastasis, chemoresistance, radioresistance, and cancer-associated thrombosis. It also summarizes three major NET-targeting strategies: enzymatic degradation, inhibition of NET formation, and indirect pharmacologic modulation, and collates emerging NET-related gene and lncRNA signatures that predict prognosis and treatment response.

Across these diverse settings, the contributions to this Research Topic converge on two translational directions. First, NETs and NET-related transcriptional programs offer measurable, clinically relevant information that can be incorporated into prognosis and patient stratification across solid and hematologic malignancies. Second, preclinical data show that dismantling NETs with DNase I or preventing their formation with PAD4, neutrophil elastase, or pathway-specific inhibitors can blunt NET-driven invasion, EMT, metastasis, and treatment resistance, and may enhance the efficacy of cytotoxic, targeted, and immune therapies. An important near-term challenge will be identifying which tumors and clinical scenarios are most likely to benefit from NET-modulating strategies and what biomarkers best capture on-target activity.

Experience from non-oncologic fields already provides proof-of-principle that NETs are therapeutically actionable in humans. In severe COVID-19, where NETs contribute to diffuse alveolar damage and immunothrombosis, clinical studies have evaluated intravenous and inhaled DNase I as adjunctive therapies to accelerate clearance of NETs, improve oxygenation, and reduce respiratory failure and thrombotic events. As mechanistic, biomarker, and preclinical therapeutic evidence in oncology continues to accumulate, exemplified by the nine articles in this Topic, the field appears increasingly close to translating NET-directed strategies into formal cancer trials. Immunogenic solid tumors with TAN-rich microenvironments, and high-risk hematologic malignancies defined by NET-related signatures are all plausible early candidates, where combining NET-modulating agents with standard and immune therapies could be guided by validated NET assays and molecular signatures.

By spanning basic mechanisms, metabolism, EMT, systems-level modeling, organ-specific disease, and supportive care, this Research Topic underscores NETs as central orchestrators of tumor microenvironments and as promising targets for risk stratification and therapeutic intervention in cancer, while highlighting concrete paths toward clinical translation.

